# KIF15 Promotes Progression of Castration Resistant Prostate Cancer by Activating EGFR Signaling Pathway

**DOI:** 10.3389/fonc.2021.679173

**Published:** 2021-11-04

**Authors:** Lin Gao, Ru Zhao, Junmei Liu, Wenbo Zhang, Feifei Sun, Qianshuo Yin, Xin Wang, Meng Wang, Tingting Feng, Yiming Qin, Wenjie Cai, Qianni Li, Hanchen Dong, Xueqing Chen, Xueting Xiong, Hui Liu, Jing Hu, Weiwen Chen, Bo Han

**Affiliations:** ^1^The Key Laboratory of Experimental Teratology, Ministry of Education and Department of Pathology, School of Basic Medical Sciences, Cheeloo College of Medicine, Shandong University, Jinan, China; ^2^Department of Biochemistry and Molecular Biology, School of Basic Medical Sciences, Cheeloo College of Medicine, Shandong University, Jinan, China; ^3^School of Basic Medical Sciences, Shandong University, Jinan, China; ^4^College of Chemical Engineering and Materials Science, Shandong Normal University, Jinan, China; ^5^Department of Molecular Genetics, University of Toronto, Toronto, ON, Canada; ^6^Department of Pathology, Qilu Hospital, Cheeloo College of Medicine, Shandong University, Jinan, China

**Keywords:** KIF15, EGFR, Cdc42, CRPC, protein stability

## Abstract

Castration-resistant prostate cancer (CRPC) continues to be a major clinical problem and its underlying mechanisms are still not fully understood. The epidermal growth factor receptor (EGFR) activation is an important event that regulates mitogenic signaling. EGFR signaling plays an important role in the transition from androgen dependence to castration-resistant state in prostate cancer (PCa). Kinesin family member 15 (KIF15) has been suggested to be overexpressed in multiple malignancies. Here, we demonstrate that KIF15 expression is elevated in CRPC. We show that KIF15 contributes to CRPC progression by enhancing the EGFR signaling pathway, which includes complex network intermediates such as mitogen-activated protein kinase (MAPK) and phosphatidylinositol 3-kinase (PI3K)/AKT pathways. In CRPC tumors, increased expression of KIF15 is positively correlated with EGFR protein level. KIF15 binds to EGFR, and prevents EGFR proteins from degradation in a Cdc42-dependent manner. These findings highlight the key role of KIF15 in the development of CRPC and rationalize KIF15 as a potential therapeutic target.

## Introduction

Prostate cancer (PCa) is the most commonly diagnosed cancer in men worldwide (1). Androgen deprivation treatment is the standard treatment for patients with advanced PCa (2). However, a more aggressive castration-resistant prostate cancer (CRPC) inevitably develops (3). Several novel therapeutic agents have been developed for CRPC, but the prognosis for patients with CRPC remains poor (4, 5). Therefore, the identification of novel therapeutic targets for CRPC is an urgent issue.

Epidermal growth factor receptor (EGFR), a member of the erbB family, regulates proliferation, differentiation, survival, and migration in multiple type of cells (6). EGFR plays an important role in the pathogenesis of PCa and in the CRPC progression (7–9). High levels of EGFR expression correlate with PCa progression (6, 7, 10, 11). EGFR usually acts at the plasma membrane or on vesicles belonging to the endosomal compartment (12); however, it can also localize to the nucleus and mitochondria (13). Epidermal growth factor (EGF) engagement activates the intrinsic kinase activity of EGFR which leads to the activation of several downstream intracellular signaling pathways, including the mitogen-activated protein kinase (MAPK) and phosphatidylinositol 3-kinase (PI3K)/AKT signaling (14, 15). These pathways can then mediate multiple physiological and pathological processes such as cell cycle progression and cell survival (16, 17). Constitutively activated MAPK and PI3K/AKT signaling occur in CRPC cells (18, 19), and they have been proposed as the important pathways in promoting PCa progression to CRPC (20).

Kinesins represent a superfamily of microtubule-dependent motor proteins that are involved in intracellular transport and mitosis (21). Kinesin family member 15 (KIF15) is an N-terminal and plus-end-directed motor that plays a critical role in the formation of bipolar spindles (22). It plays an important role in developing neuronal axons (23) and participates in the transport of macromolecules in several essential cellular processes, such as mitosis and meiosis (24). Recently, KIF15 was found to be overexpressed in several malignancies including pancreatic cancer, hepatocellular carcinoma, lung cancer, and breast cancer (25–28). Our previous study showed that KIF15 expression was elevated in enzalutamide resistant PCa, and promotes androgen receptor (AR) protein stabilization (29). KIF15 promotes cell proliferation in 22Rv1 and PC3 cells (29), which were CRPC cell lines, suggesting that KIF15 may correlate with CRPC progression. However, the function of KIF15 in CRPC cells has not been characterized.

In this study, we demonstrate that KIF15 expression is elevated in CRPC and KIF15 promotes CRPC progression. KIF15 inhibits degradation of EGFR protein in a cell division cycle 42 (Cdc42)-dependent manner, resulting in the activation of MAPK and PI3K/AKT signaling pathways. Therefore, our results highlight KIF15 as a potential novel therapeutic target for CRPC.

## Materials and Methods

### Patients

A total of 49 PCa patients participated in our study. The tumor samples were obtained from Qilu Hospital of Shandong University (Jinan, China) between 2003 and 2015. The first group included 28 men with primary PCa who have undergone radical prostatectomy without receiving preoperative radiation or androgen deprivation treatment. The second group included 21 patients with CRPC treated by transurethral resection of the prostate to relieve symptomatic obstruction due to locally advanced disease. The initial treatment for patients was either observation or surgery. Development of CRPC was treated by flutamide or bicalutamide. This study was conducted in accordance with the International Ethical Guidelines for Biomedical Research Involving Human Subjects. The study protocol was approved by the Institutional Review Board of Medicine School of Shandong University (ECSBMSSDU2019-1-021). The informed written consent was obtained from each patient.

### Immunohistochemistry

Immunohistochemistry (IHC) assays were performed as previously described using the PV9000 kit (Zsbio) (29). Slides were treated with antigen retrieval in EDTA (pH 8.0) in a pressure cooker for 10 minutes and then incubated with 3% H_2_O_2_ for 10 minutes at room temperature. Nonspecific antibody binding was blocked by subsequent incubation with goat serum (ZLI-9056; Zsbio) for 30 minutes at room temperature. Slides were then incubated overnight with anti-KIF15 (1:100, cat no. 55407-1-AP; Proteintech) or anti-EGFR (1:100, cat no. ab52894; Abcam) at 4°C. Tissues were analyzed by two independent pathologists (W.X.L. and H.B.) and KIF15 staining was scored semi-quantitatively based on cells with positive staining (0 = negative staining, 1 = weak staining, 2 = moderate staining, 3 = strong staining). For analysis, we combined both negative and weak KIF15 positive tumors into one group, and moderate and strong KIF15 positive tumors into the other. EGFR cell membrane-specific immunoreactivity was scored by estimating the percentage of positive tumor cells as previously described (7). score 0 (negative staining), no immunoreactive cell; score 1 (weak staining), positivity in 5% cancer cells; score 2 (moderate staining), positivity in 5–50% cancer cells; and score 3 (strong staining), positivity in 50% of cancer cells. Specimens were considered positive for EGFR expression (EGFR+) when the score was 2 or 3.

### Cell Culture and Reagents

LNCaP, C4-2B, and PC3 cells were purchased from American Type Culture Collection (ATCC) (Rockville, MD, USA) between 2015-2018, and cultured following ATCC’s instructions except for the indicated treatment. Cells were authenticated by short tandem repeat analysis within the last 2 years. The cumulative culture length of the cells between thawing and use in this study was less than 15 passages. All of the newly revived cells were tested free of mycoplasma contamination by Hoechst 33258 staining (Beyotime, Jiangsu, China). EGF was obtained from Peprotech (NJ, USA), 20 ng/ml EGF was used for 20 minutes in this study.

### Western Blot

Western blot assays were performed as previously described (29). Primary antibodies used in Western blot assays are anti-KIF15 (2 µg/ml, cat no. H00056992-M01; Abnova), anti-EGFR (1:1000, cat no. 4267; Cell Signaling Technology), anti-Cdc42 (1:1000, cat no. ET1701-7; HUABIO), anti-MEK (1:1000, cat no. 4694; Cell Signaling Technology), anti-p-MEK (Ser217/221) (1:1000, cat no. 9154; Cell Signaling Technology), anti-ERK (1:1000, cat no. 4695; Cell Signaling Technology), anti-p-ERK (Thr202/Tyr204) (1:1000, cat no. 4370; Cell Signaling Technology), anti-AKT (1:1000, cat no. 4685; Cell Signaling Technology), anti-p-AKT (Ser473) (1:1000, cat no. 4060; Cell Signaling Technology), anti-CDK2 (1:1000, cat no. CY5020; Abways), anti-CyclinD1 (1:1000, cat no. CY5404; Abways), anti-CyclinE1 (1:1000, cat no. CY5815; Abways), and anti-GAPDH (1:1000, cat no.ab181602; Abcam).

### Quantitative Real Time-PCR (qRT-PCR)

qRT-PCR was performed as previously described (30). The sequences of primers were as follows: *KIF15* forward, 5’-CAACCAAGTAATGAAGGTGATGCC-3’; *KIF15* reverse, 5’-AACGTGAAGGTCTTGGGCTC-3’; *EGFR* forward, 5’‐AGGCACGAGTAACAAGCTCAC‐3’; *EGFR* reverse, 5’‐ATGAGGACATAACCAGCCACC‐3’; *GAPDH* forward, 5’-GCACCGTCAAGGCTGAGAAC -3’; *GAPDH* reverse, 5’-TGGTGAAGACGCCAGTGGA-3’. *GAPDH* was included as an endogenous control. The relative expression of indicated gene was calculated using the 2^(–ΔΔCt)^ method.

### Plasmids, siRNAs and Cell Transfection

KIF15 (Gene ID: 56992; vector: PcDNA3.1) cDNA expression vectors were designed and synthesized by Sangon Biotech (Shanghai, China). SiRNAs were purchased from GenePharma (Shanghai, China). The sequences of siRNAs were: siKIF15 #1: 5’-GGACAUAAAUUGCAAAUAC-3’; siKIF15 #2: 5’-GGAACAAAUGAGUGCUCUUTT-3’; siEGFR: 5’-GUAAUUAUGUGGUGACAGATT-3’. Cdc42‐Q61L (Gene ID: 998; vector: PcDNA3.1) and Cdc42‐T17N (Gene ID: 998; vector: PcDNA3.1) cDNA expression vectors were designed and synthesized by Biosune Biotech (Shanghai, China). Lipofectamine 3000 (Invitrogen, Carlsbad, CA) was used for transfection following the manufacturer’s instruction. The effect of transfection efficiency was confirmed using qRT-PCR and Western blot assay. Lentiviral plasmids encoding shRNAs against control (NC; LV3-NC; 5’- GTTCTCCGAACGTGTCACGT -3’) and KIF15 (shKIF15; LV3-shKIF15; 5’- GGAACAAATGAGTGCTCTT -3’) were purchased from GenePharma (Shanghai, China). C4-2B cells with KIF15 knockdown were achieved by lentiviral approaches combined with puromycin selection as we reported (29).

### Cell Proliferation, Colony Formation, and Migration Assays

Cellular proliferation was measured by 3-(4,5-dimethylthiazol-2-yl)-5-(3-carboxymethoxyphenyl)-2-(4-sulfophenyl)-2H-tetrazolium inner salt (MTS) (Promega, Madison, WI, USA) and clonal formation assays. The transwell assay was used to measure the migration of PCa cells. Both assays were performed as previously described (30).

### Pulldown Assays

Rho GTPase pulldown assays were performed as previously described (31). GST-PAK-CRIB Rac/Cdc42 Isolation Kit was purchased from Kerafast (Boston, USA). The cells were lysed and centrifuged. The supernatant was transferred to new tubes containing agarose beads pre-coupled with PAK-CRIB and incubated with rotation at 4°C for 30 minutes. The beads were then washed, and the proteins bound on the beads were separated by SDS-PAGE. The amounts of active Cdc42 were determined by Western blot analysis with the corresponding antibodies.

### Tumor Xenografts

Five-week-old male nude mice were purchased from Weitonglihua Biotechnology (Beijing, China). To study the function of KIF15 in CRPC growth, a total of 6.0×10^6^ C4-2B cells expressing a control shRNA (NC) or shKIF15 mixed with matrigel (1:1) were injected subcutaneously into the mice (n = 6/group). The mice were surgically castrated when the tumors reached 100-200 mm^3^. Tumor size was measured twice a week and the tumor volume was calculated with the formula: tumor volume = 0.5 × length × width^2^. Tumor tissues were harvested and weighed after 4 weeks. All animal experiments were performed following a protocol approved by the Shandong University Animal Care Committee (Document No. LL-201602005).

### Bioinformatics Analysis

Datasets of GSE35988, GSE32269, GSE74367, and GSE2443 were downloaded from the GEO database (http://www.ncbi.nlm.nih.gov/geo). KIF15 expression in these datasets were analyzed in the groups between primary PCa and CRPC. The expressed genes of KIF15_High (top 2 KIF15 highest expression) and KIF15_Low (top 2 KIF15 lowest expression) obtained from GSE35988, GSE32269 and GSE2443 were subsequently analyzed for enrichment of biological themes using Gene Set Enrichment Analysis (GSEA) (http://software.broadinstitute.org/gsea/index.jsp).

### Statistical Analysis

Statistical analysis was carried out using GraphPad Prism 7 or SPSS 20.0 software. Statistical comparisons between groups were analyzed using two-sided Student’s *t* test. All experiments *in vitro* were performed in biological triplicate. All results are presented as the mean and the standard error of the mean. *P* < 0.05 was considered statistically significant. *, *P <*0.05; **, *P* < 0.01; ***, *P <*0.001; ****, *P <* <0.0001.

## Results

### KIF15 Expression Is Elevated in CRPC

**Figure 1:**
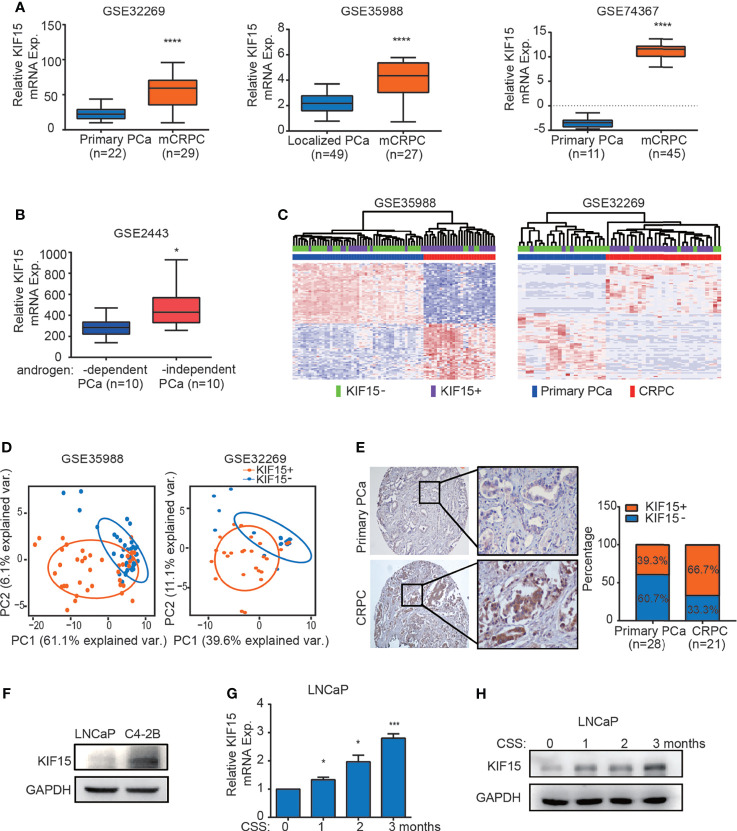
KIF15 is overexpressed in CRPC. **(A)** Expression of KIF15 in primary PCa and CRPC tissues taken from publicly available datasets of GSE32269 (left), GSE35988 (middle) and GSE74367 (right). KIF15 expression was analyzed in a) 22 samples with primary PCa from hormone-naive patients, and 29 samples with CRPC (GSE32269); b) 49 samples with localized PCa, and 27 samples with metastatic CRPC (GSE35988); c) 11 samples with primary PCa tumor and 45 samples with CRPC metastases from 32 patients (GSE74367). The statistical analysis was based on Student’s *t* test. ****P <0.0001. **(B)** KIF15 expression in androgen-dependent and independent PCa. KIF15 expression was analyzed in 10 samples with androgen-dependent primary PCa and 10 samples with androgen-independent primary PCa (GSE2443). The statistical analysis was based on Student’s *t* test. *P <0.05. **(C)** Unsupervised clustering analyses of GSE35988 (left) and GSE32269 (right) datasets based on differentially expressed genes of primary PCa and CRPC tissues. Patients statue are shown in the annotation column. Patients were categorized according to KIF15 expression or PCa risk assessment. Green: patients with low KIF15 expression (50% cutoff; KIF15-); Purple: patients with high KIF15 expression (50% cutoff; KIF15+); Blue: patients with primary PCa (Primary PCa); Red: patients with CRPC. **(D)** PCA analysis of unique CRPC-upregulated gene expression pattern between KIF15+ (high KIF15 expression, 50% cutoff) and KIF15- (low KIF15 expression, 50% cutoff) of PCa patients from GSE35988 and GSE32269 datasets. CRPC-upregulated genes were obtained from the top100 high expressed genes in CRPC compared with primary PCa in each dataset. Each point represents a patient. **(E)** The percentage of KIF15 expression distributed in PCa cases in Qilu Hospital with primary PCa or CRPC. Left panel: representative IHC images for KIF15 expression. Right panel: the percentage of KIF15 expression distributed in 49 PCa cases in Qilu Hospital with primary PCa or CRPC. **(F)** KIF15 levels in LNCaP and C4-2B cells analyzed by Western blot assay. LNCaP and C4-2B cells were harvested and whole lysates were subjected to Western blot. **(G, H)** Levels of KIF15 mRNA **(G)** and protein **(H)** in LNCaP cells with prolonged androgen-deprivation treatment. qRT-PCR and Western blot analysis were performed to detect KIF15 expression in LNCaP cells after androgen-deprived treatment in charcoal-stripped medium for the indicated time periods. CSS, Charcoal stripped fetal bovine serum; Exp., Expression; *P < 0.05; ***P < 0.001 *vs.* 0 month.

Our previous study showed that KIF15 promotes cell proliferation in androgen dependent cell line LNCaP and CRPC cell lines, including C4-2B, 22Rv1, and PC3 (29), these results suggest that KIF15 may correlate with CRPC progression. To investigate the clinicopathological significance of KIF15 expression in CRPC patients, we first analyzed the level of KIF15 using published datasets. As shown in **Figure 1A**, KIF15 expression was significantly upregulated in CRPC compared to primary PCa tissues in GSE32269 (32) (*P* < 0.0001), GSE35988 (33) (*P* < 0.0001) and GSE74367 (34) (*P* < 0.0001) datasets. Furthermore, KIF15 expression is elevated in androgen-independent than androgen-dependent PCa in GSE2443 (35) (**Figure 1B**, *P* < 0.05). In GSE35988 and GSE32269 datasets, PCa samples were divided into either the KIF15 high expression group (50% cut off) or KIF15 low expression group. Patients with high KIF15 expression were tightly clustered apart from ones with low KIF15 expression and were congruent with CRPC subgroup (**Figure 1C**). As shown in **Figure 1D**, principal component analysis (PCA) demonstrated that PCa patients with high KIF15 expression displayed an expression pattern of CRPC-upregulated genes distinct from PCa patients with low KIF15 expression. To confirm our findings, we then analyzed KIF15 expression in clinical specimens from primary PCa and CRPC cases from Qilu Hospital of Shandong University. As shown in **Figure 1E**, KIF15 is mainly expressed in the cytoplasm of tumor cells and its expression was significantly higher in CRPC samples than in primary PCa samples. Remarkably, among primary PCa cases, 17 (60.7%) showed negative or weak staining (7 cases: negative; 10 cases: weak), and only 11 (39.3%) had moderate or strong staining for KIF15 (6 cases: moderate; 5 cases: strong). However, 7 (33.3%) were negative or weak (2 cases: negative; 5 cases: weak), whereas 14 (66.7%) CRPC cases showed moderate or strong expression (6 cases: moderate; 8 cases: strong). Overall, CRPC specimens showed significantly stronger expression of KIF15 than primary PCa samples (**Figure 1E**, *P* = 0.034). Next, we evaluated the KIF15 expression in androgen-dependent LNCaP and CRPC cell lines of C4-2B. As shown in **Figure 1F**, KIF15 was dramatically upregulated in C4-2B. Notably, prolonged androgen deprivation for 3 months in LNCaP cells continuously enhanced KIF15 expression at both mRNA and protein levels (**Figures 1G, H****)**. These results indicate that enhanced KIF15 expression is highly correlated in CRPC.

### KIF15 Promotes CRPC Progression *In Vitro* and *In Vivo*

**Figure 2:**
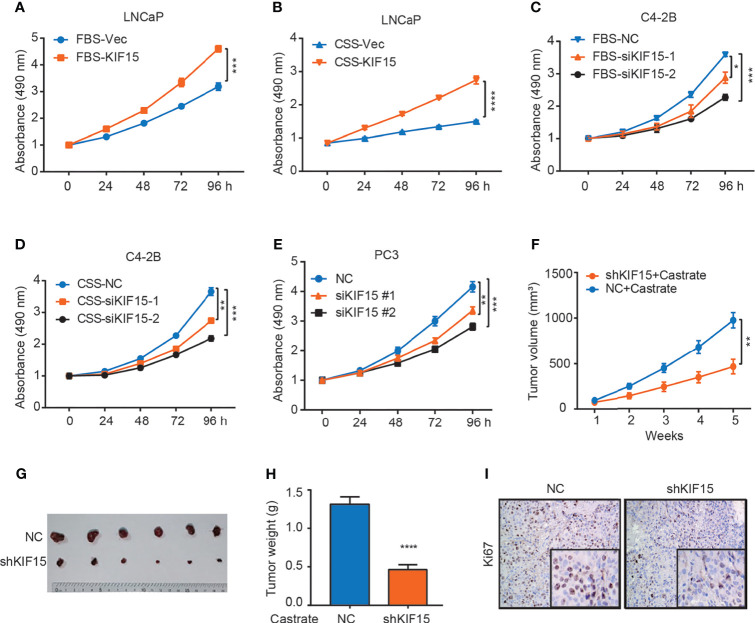
KIF15 promotes CRPC *in vitro* and *in vivo*. **(A, B)** Cell proliferation of LNCaP cells with FBS or CSS treatment assessed by MTS assays. LNCaP cells were cultured in FBS medium **(A)** or CSS medium for 48 hours **(B)**, these cells were transfected with corresponding siRNA and subjected to MTS assays. Vec, vector. ***P <0.001; ****P <0.0001. **(C, D)** Cell proliferation of C4-2B cells with FBS or CSS treatment assessed by MTS assays. C4-2B cells were cultured in FBS medium **(C)** or CSS medium for 48 hours **(D)**, these cells were transfected with corresponding siRNA and subjected to MTS assays. NC, negative control; *P <0.05; **P < 0.01; ***P <0.001. **(E)** Cell proliferation of PC3 cells assessed by MTS assays. PC3 cells were transiently transfected with corresponding siRNA, the cells were subjected to MTS assays. **P < 0.01; ***P <0.001. **(F–H)** Xenograft tumor growth after KIF15 depletion. C4-2B cells with stable KIF15 knockdown or its control were injected subcutaneously into nude mice (6 mice per group). Tumor size was measured twice every week **(F)**. At the endpoint, tumors isolated from euthanized mice were photographed **(G)** and weighed **(H)**. **P < 0.01; ****P <0.0001. **(I)** Representative images of Ki67 IHC staining of xenograft tumor derived from C4-2B NC/shKIF15 cells.

To determine whether KIF15 serves a functional role in CRPC progression, we overexpressed its expression in LNCaP cells and suppressed its expression in C4-2B cells with or without androgen depletion. We found that KIF15 greatly enhanced LNCaP cell proliferation under androgen depletion (charcoal stripped fetal bovine serum, CSS) than androgen-repletion conditions (fetal bovine serum, FBS; CSS *vs.* FBS; 1.8 folds *vs.* 1.4 folds) (**Figures 2A, B** and **Supplementary Figure S1A**). SiRNAs against KIF15 significantly reduced the total cell numbers of C4-2B under FBS as well as CSS conditions relative to their control counterparts (**Figures 2C, D** and **Supplementary Figure S1A**). Furthermore, siRNA knockdown of KIF15 in PC3 cells, an AR-negative CRPC cells (20), significantly inhibited cell proliferation (**Figure 2E****)**. Due to higher efficiency of transfection, siKIF15#2 was chosen for KIF15 knockdown in following experiments. As shown in **Supplementary Figures S1B–D**, KIF15 promoted cells migration and clone formation in both LNCaP and C4-2B cells. In addition, cell cycle distribution analysis demonstrated that silencing KIF15 could lead to a significant number of C4-2B and PC3 cells to accumulate in the G1 phase (**Supplementary Figure S1E**). Furthermore, KIF15 depletion of C4-2B xenografts in castrated nude mice resulted in delayed tumor progression; the mean tumor volume 463.5 ± 79.92 mm^3^ in C4-2B shKIF15 xenografts while it was 979 ± 84.57 mm^3^ in the control group (*P* = 0.001) (the weight of tumors; shKIF15 *vs.* control; 0.4633 ± 0.06312 g *vs.* 1.313 ± 0.09698 g; *P* < 0.0001) (**Figures 2F–H**). Moreover, the Ki67 percentage score of tumor cells in the shKIF15 group was relatively low when compared to cells in the NC group (**Figure 2I**). Collectively, our data suggested that KIF15 is required for the proliferation of CRPC cells.

### KIF15 Regulates EGFR Signaling in CRPC Cells

**Figure 3:**
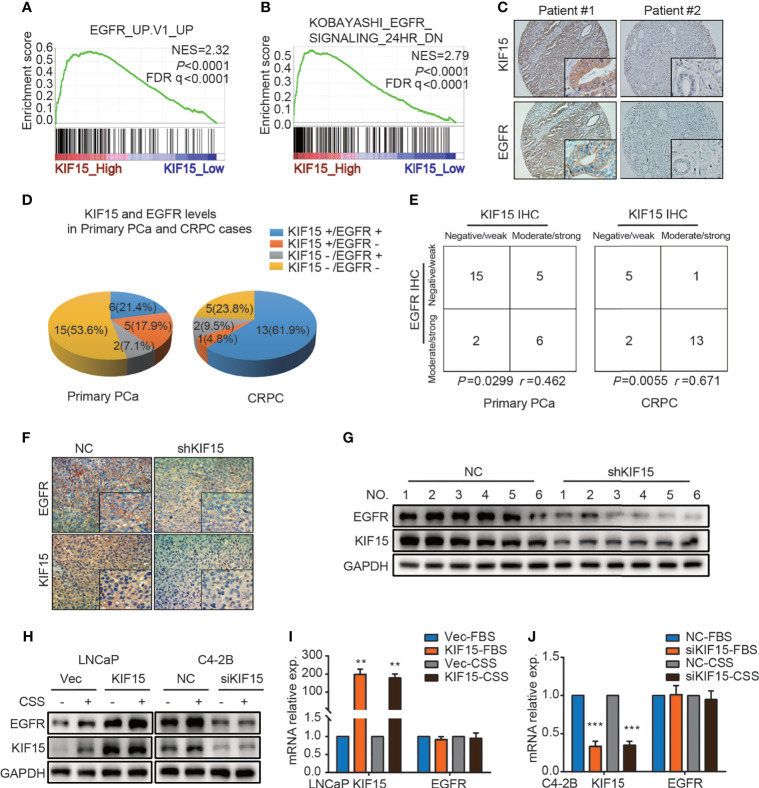
KIF15 promotes EGFR expression in PCa. **(A)** GSEA analysis of EGFR signatures (genes upregulated upon EGFR elevated) from a microarray dataset (GSE35988) that profiled CRPC cases with KIF15_High (the highest 2 samples) or KIF15_Low (the lowest 2 samples) expression. NES = 2.32; *P* < 0.0001, FDR q < 0.0001. **(B)** GSEA analysis of EGFR signatures (down-regulated after treatment with EGFR inhibitor) from a microarray dataset (GSE32269) that profiled CRPC cases with KIF15_High (the highest 2 samples) or KIF15_Low (the lowest 2 samples) expression. NES = 2.79; *P* < 0.0001, FDR q < 0.0001. **(C, D)** Representative IHC images **(C)** and quantitative analysis **(D)** for EGFR and KIF15 expression in PCa cases in Qilu Hospital. KIF15-, negative and weak KIF15 positive tumors examined by IHC staining; KIF15+, moderate and strong KIF15 positive tumors examined by IHC staining; EGFR-, negative and weak EGFR positive tumors examined by IHC staining; EGFR+, moderate and strong EGFR positive tumors examined by IHC staining. **(E)** Contingency table for KIF15 expression and EGFR status by IHC in primary PCa cases and CRPC cases in Qilu Hospital. **(F, G)** EGFR protein expression in C4-2B xenograft examined by IHC staining **(F)** and Western blot **(G)**. **(H)** EGFR protein expression in LNCaP cells (left) and C4-2B (right) cells. These cells were cultured and passaged in CSS medium or FBS medium for 1 month, and then they were transfected with corresponding expression plasmids for 48 hours or siRNA for 72 hours. Then cells were collected, lysed for Western blot assay. **(I, J)** The relative mRNA expression of KIF15 and EGFR in LNCaP **(I)** and C4-2B **(J)** cells with indicated treatment. LNCaP **(I)** and C4-2B **(J)** cells with indicated treatment as **(H)** were transfected with expression plasmids or siRNAs for 48 hours. The total RNA was extracted, and the mRNA levels of KIF15 and EGFR were determined by qRT-PCR. **P < 0.01; ***P <0.001.

EGFR signaling plays an important role in the progression of PCa and the transformation to CRPC (7). We firstly utilized multiple public datasets to characterize the relationship between EGFR and KIF15 in CRPC progression. As shown in **Figure 3A**, GSEA was performed using microarray datasets from GEO database (GSE35988) and revealed that genes positively related to EGFR were enriched in the KIF15_High samples (NES = 2.32; *P* < 0.0001; FDR q< 0.0001). Additionally, genes down-regulated after treatment with EGFR inhibitor were enriched in the KIF15_High samples, which were analyzed by GSEA using GSE32269 (NES = 2.79; *P* < 0.0001; FDR q< 0.0001) as well as in GSE2443 (NES = 2.57; *P* < 0.0001; FDR q< 0.0001) microarray data (**Figure 3B** and **Supplementary Figure S2A****)**. These results suggested that EGFR pathway was positively related with KIF15 high expression in PCa. Importantly, 6 out of 28 (21.4%) primary PCa cases from Qilu Hospital showed KIF15+/EGFR+ by IHC staining (moderate or strong staining for both KIF15 and EGFR). Accordingly, 15 out of 28 (53.6%) cases were KIF15-/EGFR- by IHC staining (negative or weak staining for both KIF15 and EGFR). By contrast, only 5 (17.9%) cases demonstrated KIF15+/EGFR- (moderate or strong staining for KIF15 whereas negative or weak staining for EGFR) and 2 (7.1%) cases demonstrated KIF15-/EGFR+ (negative or weak staining for KIF15 and moderate or strong staining for EGFR). Among CRPC cases, IHC staining showed that 13 (61.9%) cases were KIF15+/EGFR+, 5 (23.8%) cases with KIF15-/EGFR-. By contrast, only 1 (4.8%) case was KIF15+/EGFR- and 2 (9.5%) cases were KIF15-/EGFR+ (**Figures 3C, D****)**. A positive correlation of KIF15 and EGFR expression was observed in primary PCa cases (*P* = 0.0299, *r* = 0.462) and CRPC cases (*P* = 0.0055, *r* = 0.671) (**Figure 3E**). Overall, these data suggest that high protein levels of KIF15 correlated with increased EGFR protein levels in prostate tumor samples. In addition, EGFR protein expression was significantly downregulated in C4-2B xenografts with KIF15 depletion as shown in **Figures 3F, G**. Then, we monitored EGFR expression after KIF15 overexpression in LNCaP cells, and KIF15 depletion in C4-2B and PC3 cells. As shown in **Figure 3H** and **Supplementary Figure S2B**, KIF15 overexpression enhanced EGFR protein levels in LNCaP cells, while KIF15 depletion reduced EGFR protein levels in C4-2B and PC3 cells. These effects were more significantly in CSS condition in these cells. However, EGFR mRNA levels were not altered even though these cells showed marked increases or reductions in KIF15 expression (**Figures 3I, J** and **Supplementary Figure S2C**). Together, our results suggest that KIF15 may regulate EGFR protein levels, especially in the androgen deprivation condition.

**Figure 4:**
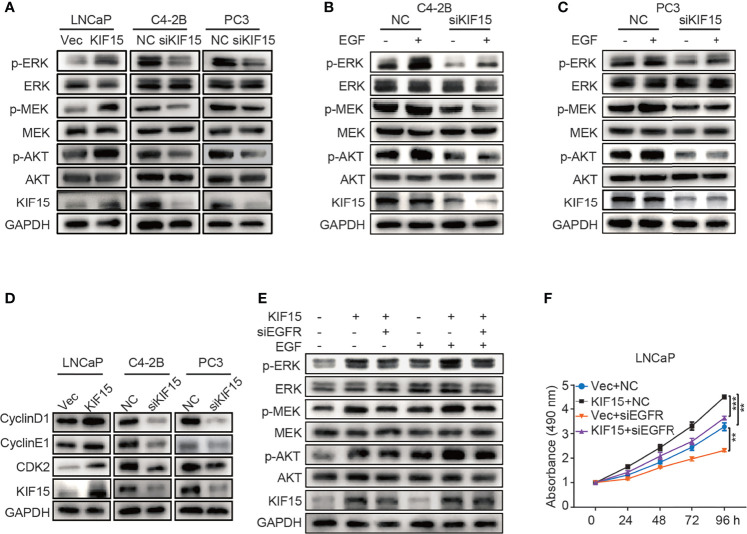
KIF15 activates EGFR signaling in CRPC cells. **(A-C)** Expression of the key molecules in MAPK and PI3K-AKT signaling pathways determined by Western blot in KIF15-overexpressed or depleted PCa cells with or without 20 ng/ml EGF treatment for 20 minutes. **(D)** The expression of cell cycle-related proteins, CyclinD1, CyclinE1, and CDK2 determined by Western blot in the indicated PCa cells with KIF15 overexpression or depletion. **(E)** Expression of the key molecules in MAPK and PI3K-AKT signaling pathways determined by Western blot in KIF15 overexpressed and EGFR depleted LNCaP cells with or without 20 ng/ml EGF treatment for 20 minutes. **(F)** Cell proliferation of LNCaP cells assessed by MTS assays. LNCaP cells were transiently transfected with the indicated expression plasmids and/or siRNA, and cell proliferation was assessed by MTS assays. **P < 0.01; ***P <0.001.

To examine how KIF15 regulates EGFR signaling, we tested the key molecules in MAPK and PI3K-AKT signaling pathways which were reported as downstream of EGFR (19, 36). As shown in **Figure 4A**, KIF15 overexpression significantly increased p-MEK, p-ERK, and p-AKT protein levels in LNCaP cells, while KIF15 knockdown reduced p-MEK, p-ERK and p-AKT levels in C4-2B and PC3 cells. However, these effects of KIF15 were more significant with EGF treatment in the corresponding cells (**Figures 4B, C****)**. Since KIF15-depleted cells showed attenuation in MAPK and AKT activity and accumulation at the G1 phase, we examined the expression of cell cycle regulatory proteins (37) in these PCa cell lines. As shown in **Figure 4D**, KIF15 knockdown significantly reduced CyclinD1, CyclinE1, and CDK2 protein levels in both C4-2B and PC3 cells, while KIF15 overexpression increased their levels in LNCaP cells. Importantly, EGFR depletion reversed KIF15 overexpression-induced activation of MEK, ERK, and AKT, and cell proliferation in LNCaP cells (**Figures 4E, F** and **Supplementary Figure S2D**). These data demonstrated that KIF15 promotes CRPC progression by activating EGFR signaling pathway.

### KIF15 Inhibits Degradation of EGFR in CRPC Cells

**Figure 5:**
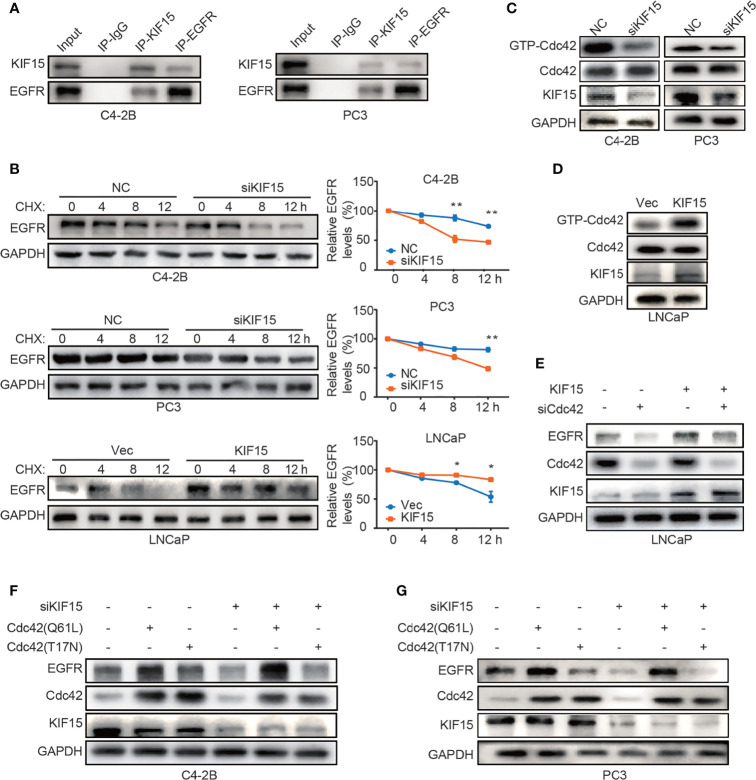
KIF15 forms a protein complex with EGFR and inhibits EGFR degradation through Cdc42 in CRPC cells. **(A)** Co-IP assays performed to detect the interaction between KIF15 and EGFR in C4-2B and PC3 cells. Protein lysis was collected from C4-2B and PC3 cells to perform co-IP with control IgG or KIF15 or EGFR antibody, followed by Western blot with indicated antibodies. **(B)** EGFR protein levels determined by immunoblotting. C4-2B (top), PC3 (middle), and LNCaP (bottom) cells were transfected with KIF15 siRNA or KIF15 expression plasmids as indicated. At 24 hours post transfection, cells were then treated with 10 μg/ml CHX and collected at 0, 4, 8, and 12 hours. Western blot assays were performed to analyze EGFR protein levels. *P <0.05; **P < 0.01. **(C, D)** GTP-Cdc42 levels analyzed by Western blot in C4-2B, PC3 **(C)**, and LNCaP **(D)** cells with the indicated treatment. C4-2B, PC3, and LNCaP cells were transfected with KIF15 siRNA or KIF15 expression plasmids as indicated. Rho GTPase pulldown assays were performed, and activated Cdc42 (GTP-Cdc42) was measured by Western blot assay. **(E–G)** EGFR protein expression in LNCaP **(E)**, C4-2B **(F)**, and PC3 **(G)** cells with the indicated treatment. LNCaP cells were transfected with KIF15 expression plasmids and siRNA against Cdc42 **(E)**, while C4-2B **(F)**, and PC3 **(G)** cells were transfected with siRNA against KIF15 and Cdc42(T17N) or Cdc42(Q61L) expression plasmids for 48 hours, then were harvested and lysed for Western blot assay.

To explore the mechanism by which KIF15 modulates EGFR protein level, we performed co-immunoprecipitation (co-IP) assays in both C4-2B and PC3 cells. As shown in **Figure 5A**, KIF15 binds to EGFR in these two cell lines. Since KIF15 regulated EGFR expression at protein levels but not at mRNA levels, we tested whether KIF15 affects EGFR protein degradation in PCa cells. C4-2B, PC3, and LNCaP cells were treated with cycloheximide (CHX) to block *de novo* protein synthesis, and EGFR protein level was analyzed by Western blot. The results showed that knockdown of KIF15 remarkably accelerates the degradation of EGFR proteins in comparison to the control cells, and KIF15 overexpression inhibited EGFR protein degradation (**Figure 5B**). These data suggest that KIF15 can stabilize the EGFR protein. It has been previously reported that Cdc42, a member of Rho GTPase family protein and a key regulator of the actin cytoskeleton, plays an important role in the process of internalization and degradation of receptors (38). Cdc42 activity is controlled by exchanging between the inactive GDP-bound form (Cdc42^GDP^) and active GTP-bound form (Cdc42^GTP^) (39). Unlike Ras, which is activated primarily by point mutations that impair its GTPase activity in human cancers, Cdc42 is activated by changes in upstream regulators. Hirsch et al. showed that EGFR protein degradation is correlated with activation of Cdc42 (40). To demonstrate the potential mechanism of KIF15 silencing-induced EGFR degradation, we tested Cdc42 activity by pulldown assays in C4-2B and PC3 cells transfected with siRNA against KIF15 and in LNCaP cells transfected with KIF15 expression plasmids. As shown in **Figures 5C, D**, Cdc42 activity was significantly attenuated in KIF15-depleted C4-2B and PC3 cells, and enhanced in LNCaP cells with KIF15 overexpression. Furthermore, Cdc42 knockdown reversed KIF15 overexpression-induced EGFR protein upregulation in LNCaP cells (**Figure 5E**). Cdc42‐Q61L was a form of Cdc42-active mutant (41), and Cdc42‐T17N was a form of Cdc42-inactive mutant (42, 43). EGFR protein level was elevated when Cdc42‐Q61L but not Cdc42‐T17N plasmids were transfected into the KIF15-depleted C4-2B and PC3 cells (**Figures 5F, G****)**. Our data supports that KIF15 inhibits degradation of EGFR in a Cdc42-dependent manner.

**Figure 6:**
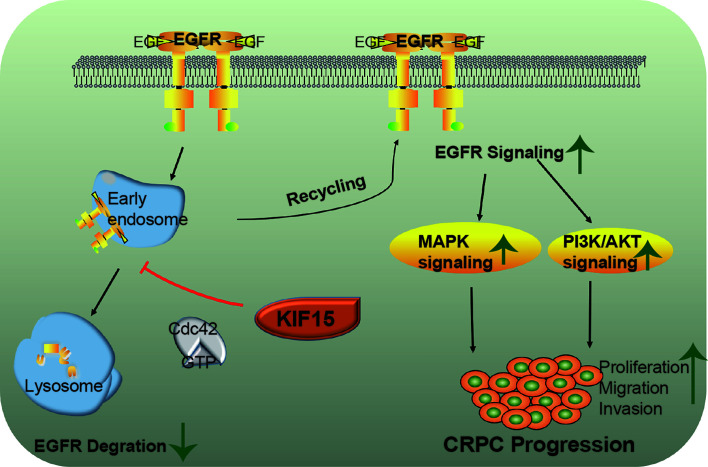
A putative model illustrating the role of KIF15 in up-regulation of EGFR signaling to promote CRPC. EGFR is ubiquitinated by EGF stimulation and sorted to the endosome, resulting in its degradation in lysosomes. KIF15 blocks EGFR from undergoing lysosomal degradation by activating Cdc42 and increases EGFR recycling back to the cell membrane. The MAPK/ERK and PI3K/AKT signaling pathways are activated, both of which can promote the proliferation and invasion of CRPC cells.

Together, KIF15 stabilizes EGFR in a Cdc42-dependent manner and activates EGFR signaling pathways to promote CRPC progression. A schematic diagram was shown in **Figure 6**.

## Discussion

Our studies reveal a novel role of KIF15 that promotes CRPC by activating EGFR signaling pathway in both AR-positive and AR-negative cells. We show that KIF15 is elevated in CRPC cells, and that there is a positive correlation between KIF15 expression and EGFR protein expression levels. Upregulation of EGFR by KIF15 is mediated *via* a transcription-independent mechanism that involves inhibiting EGFR protein degradation. KIF15 overexpression induced the activation of ERK, MEK, and AKT, which are important molecules downstream of EGFR signaling. Our results indicate that, at least in part, KIF15 promotes CRPC cell proliferation *via* EGFR-dependent signaling, which highlights a novel and prominent role of KIF15 in contributing to CRPC progression. In addition, our previous studies showed that KIF15 promotes PCa progression by increasing AR protein levels (29). These new findings reveal the extensive role of KIF15 in the progression of CRPC especially in these AR-negative cells.

Although PCa is initially androgen sensitive and responds to androgen deprivation therapies, adaptive survival pathways culminate and CRPC inevitably develops. Multiple mechanisms contribute to the progression to CRPC, including both AR-dependent (44) and AR-independent pathways (45). Although AR is an important driver of CRPC progression (44), the PI3K−AKT−mTOR pathway, Src signaling pathway and growth factor pathways, which are AR-independent pathways, also play a crucial role in CRPC (7, 18, 46, 47). Thus, novel therapies beside AR inhibition occupy an increasingly important role in the treatment of CRPC (5). Our previous studies showed that KIF15 promotes AR protein stabilization by enhancing the interaction between USP14 and AR in C4-2B enzalutamide resistant cells and 22Rv1 cells (29), suggesting that KIF15 may promote enzalutamide resistance *via* the AR pathway. In the current study, we focus on the role of KIF15 in CRPC progression and found that KIF15 could enhance the EGFR signaling pathway in both AR positive and negative cells.

Our new findings in this study that KIF15 activates EGFR signaling demonstrates that KIF15 may expedite CRPC progression by multiple pathways including AR-dependent and independent pathways. Multiple studies have highlighted the key role of AR signaling pathway in CRPC. Reactivation of AR signaling is sufficient and necessary to trigger the CRPC phenotype (48, 49). Therefore, although activation of AR and EGFR by KIF15 may simultaneously exist in AR positive CRPC cells, activation of EGFR signaling might act as a collaborative pathway. However, in AR negative CRPC cells, KIF15 may promote tumor cell proliferation through activating EGFR signaling pathway. One limitation of this study is how PTEN status affects KIF15-EGFR axis. LNCaP and C4-2B cells are PTEN-null and exhibit constitutively activated PI3K, whereas 22Rv1 cells express wild-type PTEN. In the current study, we found that KIF15 could affect EGFR protein expression both in LNCaP, C4-2B as well as in 22Rv1 (**Supplementary Figure S2E**) cells. These data suggested that PTEN status might not affect KIF15-EGFR axis in PCa cells.

Our study showed that AR-induced gene KIF15 is highly expressed in CRPC, which is consistent with the previous study (50). KIF15 expression pattern in PCa is similar with that of COBLL1, an AR-induced gene, which is highly induced in androgen-deprived cells (51). Our explanation of the mechanisms is as follows: 1) Androgen deprivation may result in reactivation of AR. In this setting, AR is high-sensitive to low androgen. Hypersensitive AR activity is correlated with upregulation of AR-binding genes. As another possibility for inducing AR-binding genes in CRPC cells, previous studies have reported the reprogramming of AR downstream genes (52, 53). 2) Some key genes related with PCa progression are upregulated in androgen-deprived condition, including OCT1 (50), ANCCA (54), and B7-H3 (55). These genes have been reported to regulate KIF15 expression in solid tumors (25, 50, 56) as well as in androgen-deprived condition. 3) Androgen deprivation is associated with pro-inflammatory in PCa (57), and KIF15 has been reported to be upregulated in inflammatory microenvironment (58). Overall, KIF15 may be an important AR-induced gene in the transition from hormone sensitive PCa to CRPC cells.

Di Lorenzo et al. and Jathal et al. have revealed that high EGFR protein correlates with PCa progression and CRPC state (7, 11). There are many mechanisms of EGFR to promote CRPC. The first is the “cross-talk” between EGFR and AR pathways in CRPC cells (59). EGFR enhances AR stability and transcriptional function, and may contribute to AR activity in CRPC (60, 61). Combining the results from our previous studies that KIF15 promotes AR protein stabilization, our new findings in this study suggest that KIF15 may regulate AR not only directly but also in an EGFR-dependent way. The second mechanism involves MAPK and PI3K/AKT pathways activation by EGFR to sustain the growth, survival, invasion and metastasis of CRPC (6). EGFR-stimulated ERK activation is required for the induction and maintenance of the increased expression of Cyclin D1, and ensures G1 phase progression in the cell-cycle (6). As one of the chromokinesin family members, KIF15 is well known for its role in regulating mitotic spindle microtubules to promote cell mitosis (22). Therefore, KIF15 may expedite the cell cycle by accelerating mitosis and by activating ERK to accelerate entrance into the S phase in interphase. Together, our results demonstrate that KIF15 can utilize multiple pathways to expedite CRPC progression.

The degradation of EGFR is regulated by multiple factors. After EGFR binds to ligands, it undergoes dimerization, autophosphorylation, and internalization. Phosphorylated EGFR is ubiquitinated and degraded in lysosomes or recycled back to the cell surface (38, 62). Our findings show that function of KIF15 reduces EGFR degradation by activating Cdc42 in PCa cells. Cdc42 is a ras-related GTP-binding protein that serves as a molecular switch in cells and directs a wide range of cellular processes and signaling activities. Activated Cdc42, through an interaction with its target/effector, inhibits the binding of c-Cbl (E3 ubiquitination ligase of EGFR) (63) to EGFR and thus prevents c-Cbl from catalyzing receptor ubiquitination. KIF15 promotes EGFR protein stabilization by activating Cdc42, whose mode of action is different from STAP-2, δ-Catenin, and UCHL1 inhibition of EGFR lysosomal degradation by competitive inhibition c-Cbl–mediated ubiquitination or abrogation of EGFR ubiquitination (64–66). These findings also posed additional questions if KIF15 can modulate other proteins in addition to EGFR in a Cdc42-dependent manner to promote CRPC. These implications warrant further investigations.

In conclusion, we further explored the functional role of KIF15 in CRPC and investigated the potential mechanisms of KIF15 in EGFR-mediated development of CRPC. Our findings uncovered that KIF15 inhibition may be considered as a potential novel strategy in CRPC patients.

## Data Availability Statement

The datasets presented in this study can be found in online repositories. The names of the repository/repositories and accession number(s) can be found in the article/**Supplementary Material**.

## Ethics Statement

The studies involving human participants were reviewed and approved by Institutional Review Board of Medicine School of Shandong University (ECSBMSSDU2019-1-021). The patients/participants provided their written informed consent to participate in this study. The animal study was reviewed and approved by Shandong University Animal Care Committee (Document No. LL-201602005).

## Author Contributions

LG and RZ contributed to the conception and design of the study as well as data acquisition and interpretation. LG drafted the manuscript. JL, WZ, XW, FS, HL and JH extracted the information from the databases. TF, MW, QL, HD, QY, YQ, WJC and XC contributed to development of methodology. XX, WWC, and BH reviewed the manuscript critically. BH supervised and designed the study. All authors contributed to the article and approved the submitted version.

## Funding

This work was supported by the National Natural Science Foundation of China (Grant No. 81972416, No. 82172818), the National Key Research And Development Program of China (No. 2018YFC0114703). Joint Research Fund of Natural Science, Shandong Province (ZR2019LZL014), and the program for outstanding PhD candidate (To TF) of Shandong University.

## Conflict of Interest

The authors declare that the research was conducted in the absence of any commercial or financial relationships that could be construed as a potential conflict of interest.

## Publisher’s Note

All claims expressed in this article are solely those of the authors and do not necessarily represent those of their affiliated organizations, or those of the publisher, the editors and the reviewers. Any product that may be evaluated in this article, or claim that may be made by its manufacturer, is not guaranteed or endorsed by the publisher.

## Supplementary Material

Supplementary Figure 1 KIF15 promotes proliferation and migration of prostate cancer cells *in vitro*. **(A)** Western blot analysis of KIF15 protein levels in indicated PCa cells. LNCaP and C4-2B cells were cultured in FBS (RPMI-1640 supplemented with 10% fetal bovine serum) medium or CSS (phenol red-free RPMI-1640 supplemented with 10% charcoal-stripped fetal bovine serum) medium for 48 hours. These cells were transfected with KIF15 expression plasmid for 48 hours (LNCaP cells) or corresponding siRNA for 72 hours (C4-2B cells), then were collected and lysed for Western blot assay. Vec, empty vector; NC, negative control. **(B)** Migration ability of indicated cells determined by transwell assays. Left panel: representative images of cell migration. Right panel: quantitative results of migration assays from triplicate experiments. **P* <0.05, ***P* < 0.01. **(C, D)** Cell proliferation determined by colony formation assays. Data shown are means ± SEM of triplicate wells and are representative of at least three replicate experiments. Comparisons between groups were analyzed using t‐tests (two‐sided). **P* <0.05, ***P* < 0.01. **(E)** Effects of KIF15 on the cell cycle determined by flow cytometry. Percentage of cells in G1, S and G2 phases are shown. C4-2B and PC3 cells were transfected with corresponding siRNA for 48 hours, then were harvested, and stained with propidium iodide dye for flow cytometric analysis of cell cycle distribution.

Supplementary Figure 2 Relationship between KIF15 and EGFR level in prostate cancer cells. **(A)** GSEA analysis of EGFR signatures (down-regulated after treatment with EGFR inhibitor) from a microarray dataset (GSE2443) that profiled with KIF15_High (the highest 2 samples) or KIF15_Low (the lowest 2 samples) expression. NES = 2.57; *P* < 0.0001, FDR q < 0.0001. **(B, C)** The protein **(B)** and mRNA **(C)** expression of EGFR determined by Western blot and qRT-PCR analysis after KIF15 transient knockdown in PC3 cells. PC3 cells were transfected with corresponding siRNA for 72 hours, then were harvested and lysed for Western blot assay **(B)**. PC3 cells were transfected with corresponding siRNA for 48 hours. The total RNA was extracted, and the mRNA levels of KIF15 and EGFR were then determined by qRT-PCR **(C)**. **P* <0.05, ***P* < 0.01, ****P* <0.001. **(D)** EGFR and KIF15 protein expression levels examined by Western blot in LNCaP cells. LNCaP cells were transiently transfected with the indicated expression plasmids and/or siRNA for 48 hours. The cells were then harvested and lysed for Western blot assay. **(E)** EGFR and KIF15 protein expression levels examined by Western blot after KIF15 siRNA knockdown in 22Rv1 cells. 22Rv1 cells were transiently transfected with corresponding siRNA for 72 hours. The cells were then harvested and lysed for Western blot assay.
