# Supplementary material for: KIF15 Promotes Progression of Castration Resistant Prostate Cancer by Activating EGFR Signaling Pathway
Source: Front Oncol. 2021 Nov 4;11:679173. doi: 10.3389/fonc.2021.679173 (PMC8599584; doi:10.3389/fonc.2021.679173)
Supplement: Supplementary file 1 [file DataSheet_1.pdf]

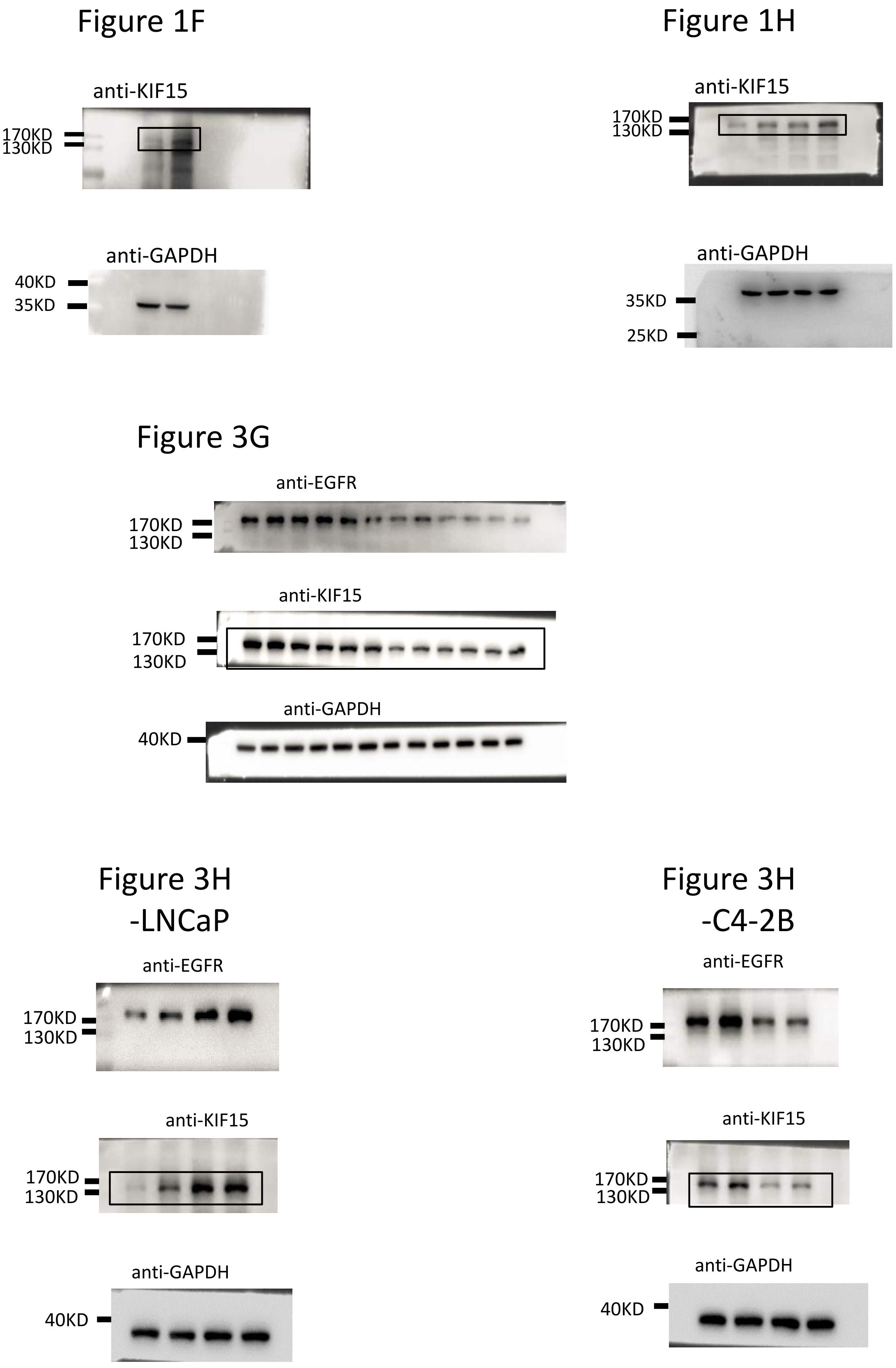

Supplementary Fig. S3. Unprocessed blot images for the western results in Fig. 1 and 3.

Figure 4A

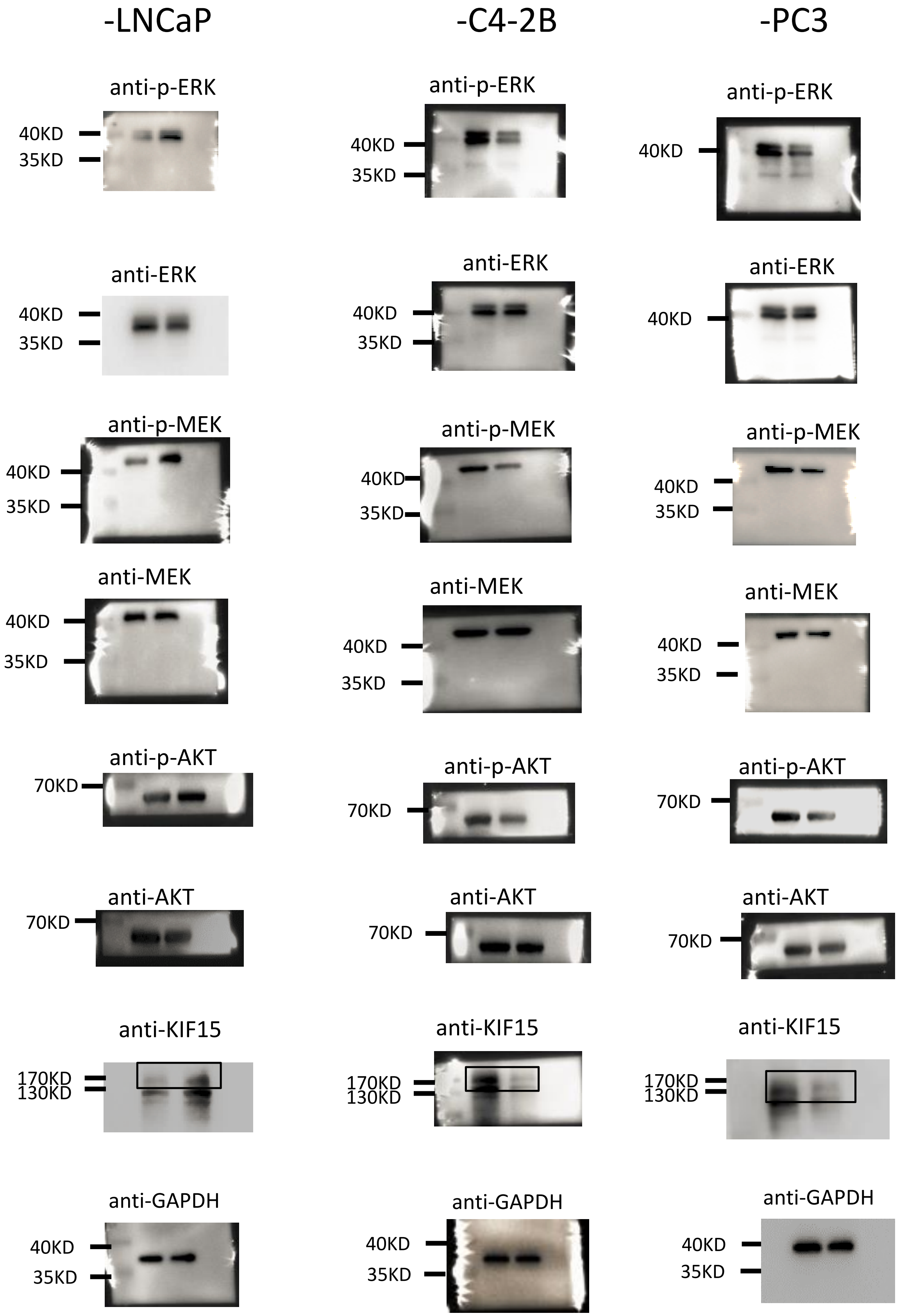

Supplementary Fig. S4. Unprocessed blot images for the western results in Fig. 4A.

Figure 4B

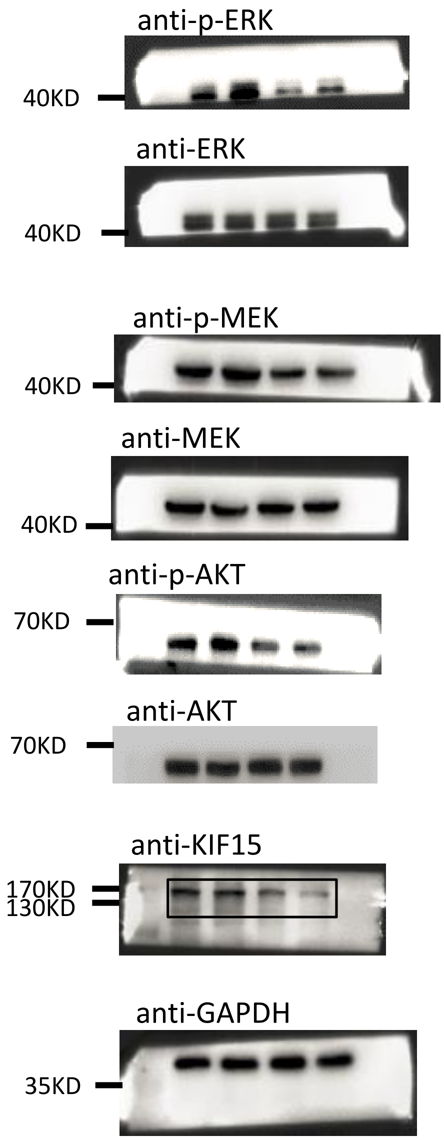

Figure 4C

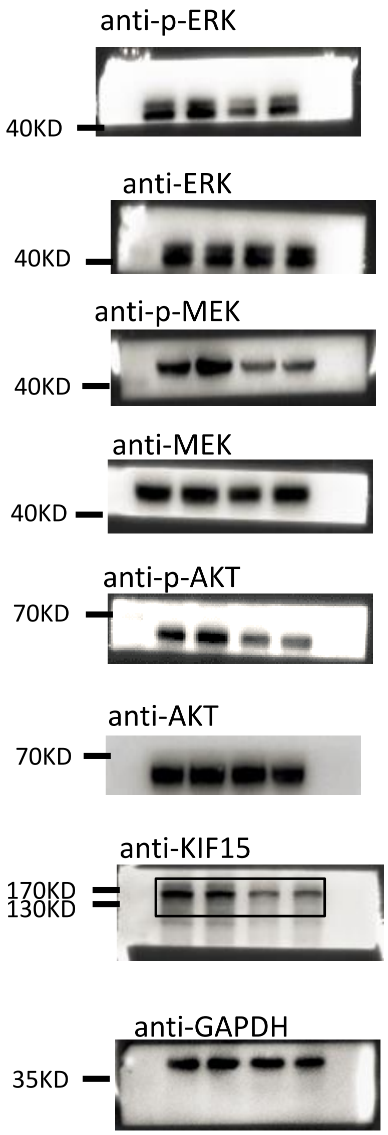

Supplementary Fig. S5. Unprocessed blot images for the western results in Fig. 4B-C.

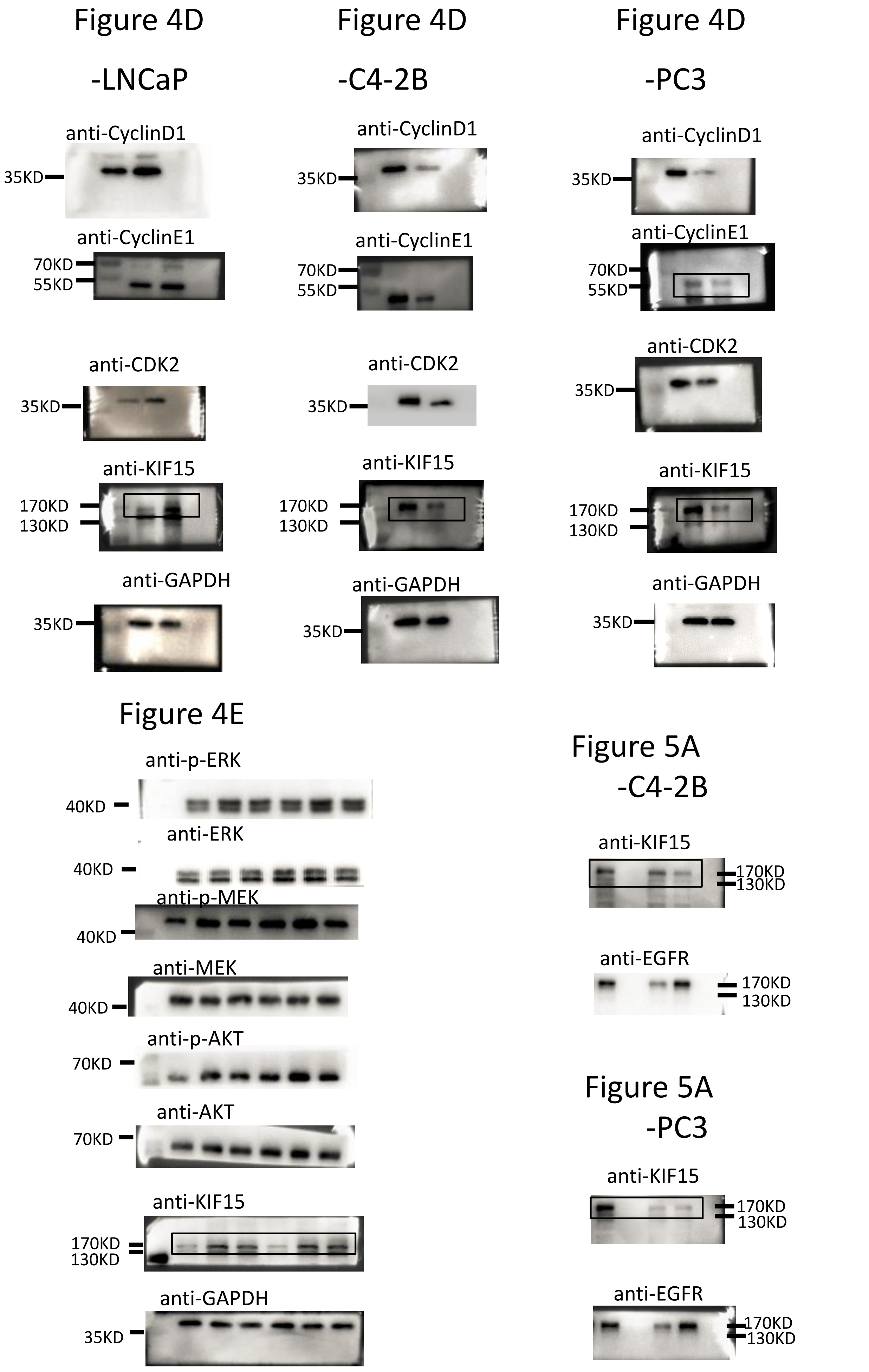

Supplementary Fig. S6. Unprocessed blot images for the western results in Fig. 4D-E and 5A.

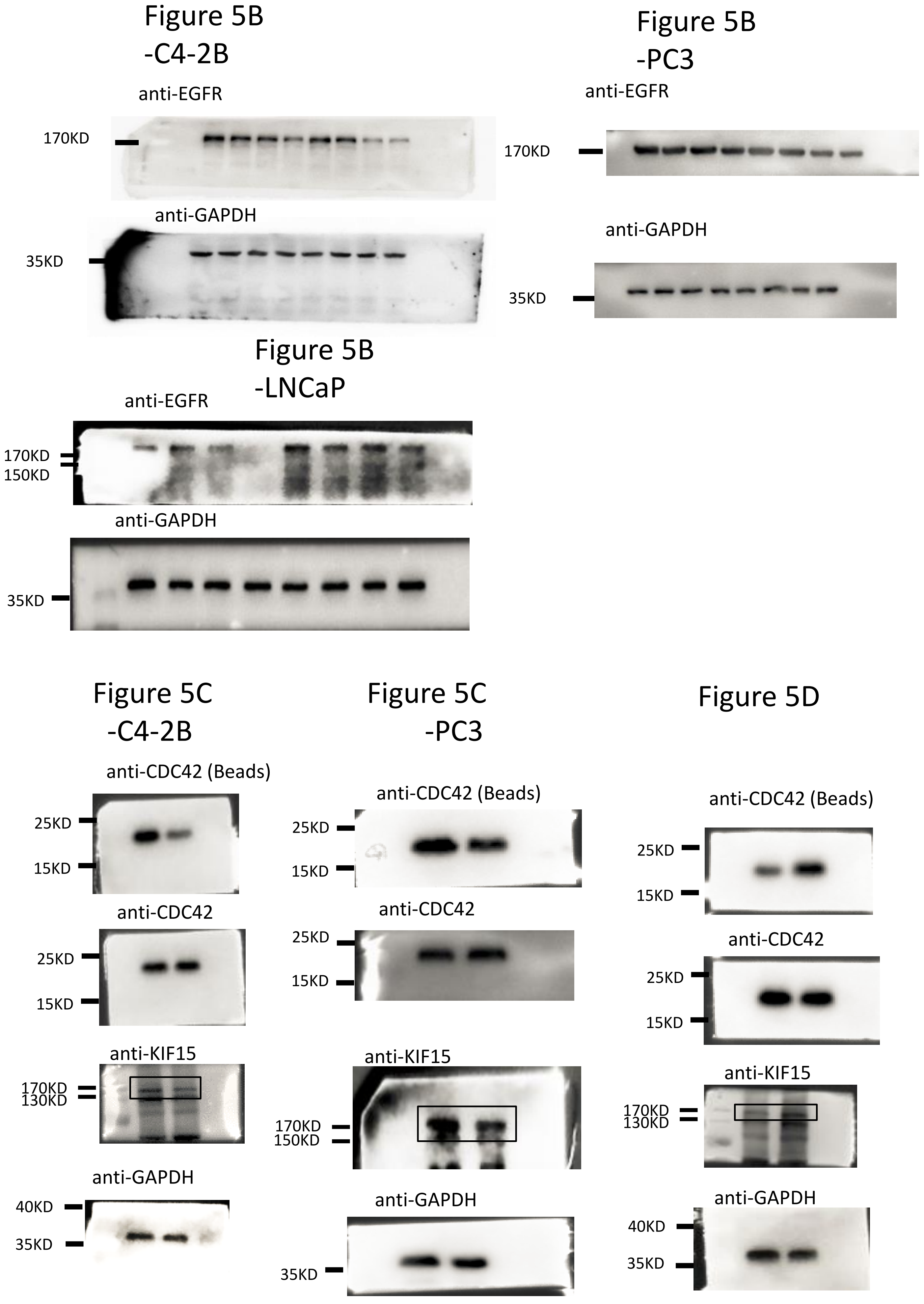

Supplementary Fig. S7. Unprocessed blot images for the western results in Fig. 5B-D.

Figure 5E

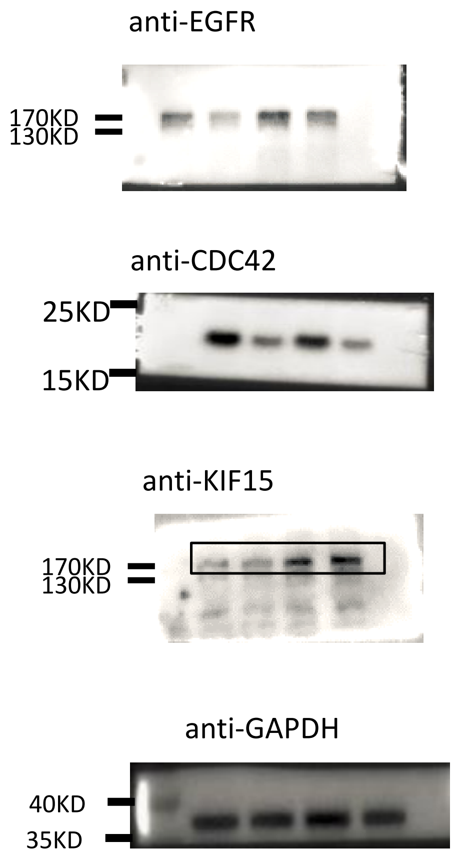

Figure 5F

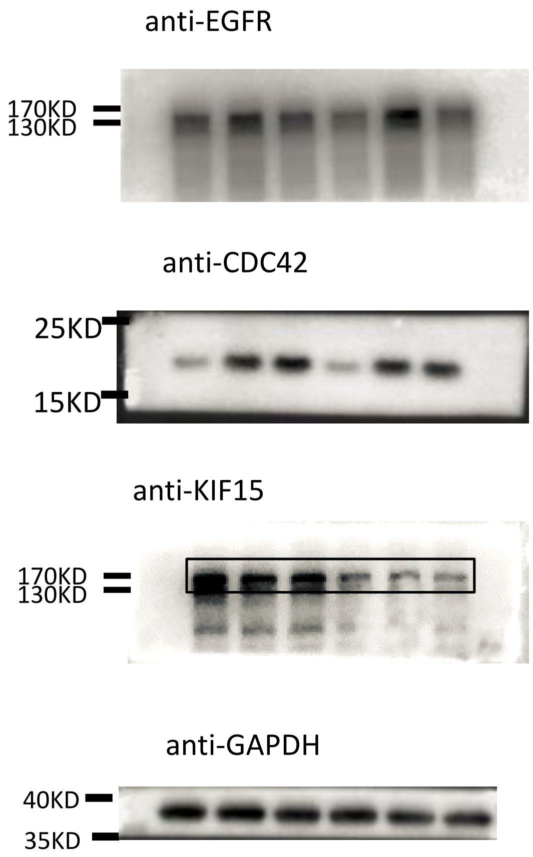

Figure 5G

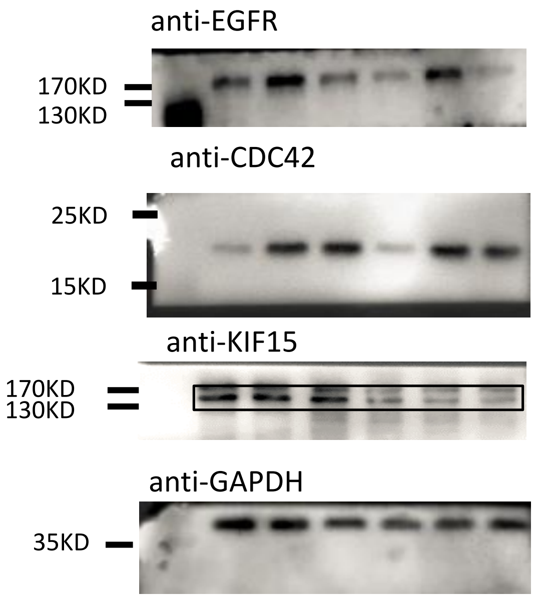

Supplementary Fig. S8. Unprocessed blot images for the western results in Fig. 5E-G.

Supplemental Figure 1A

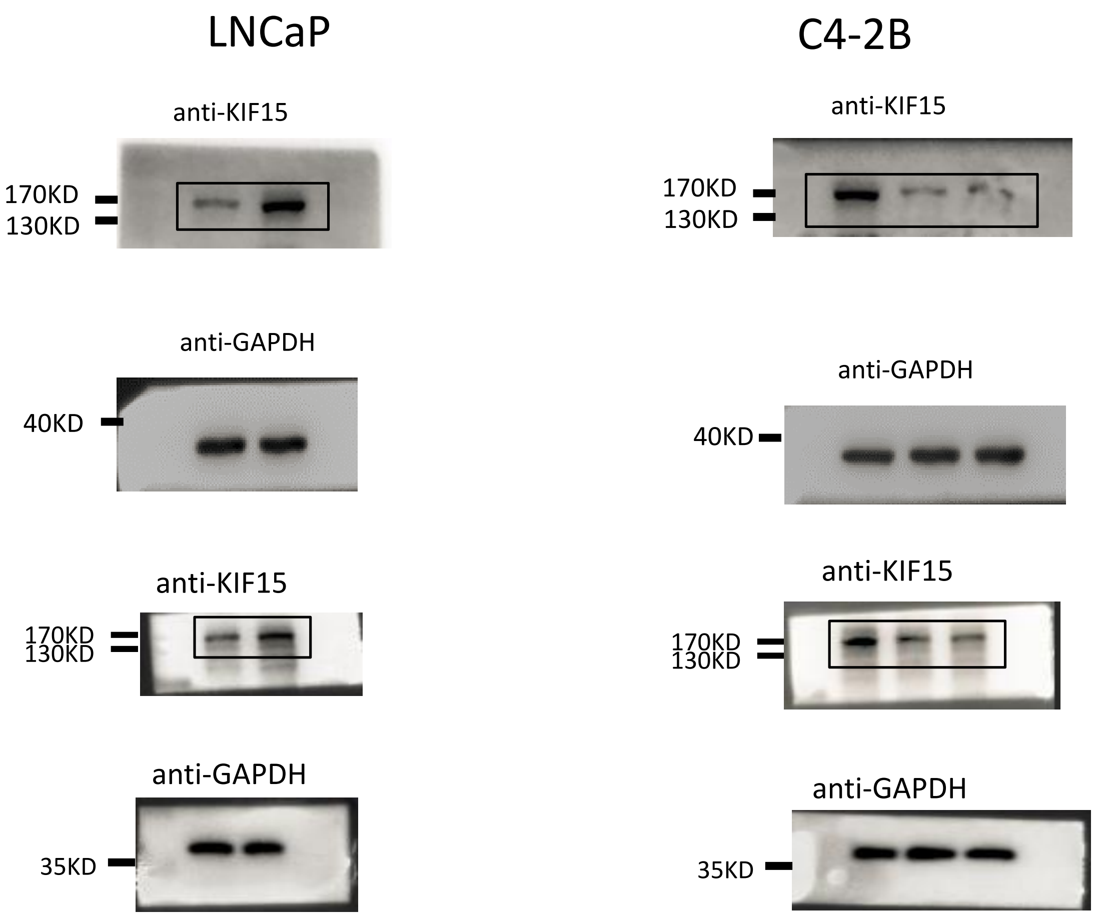

Supplemental Figure 2B

Supplemental Figure 2D

Supplemental Figure 2E

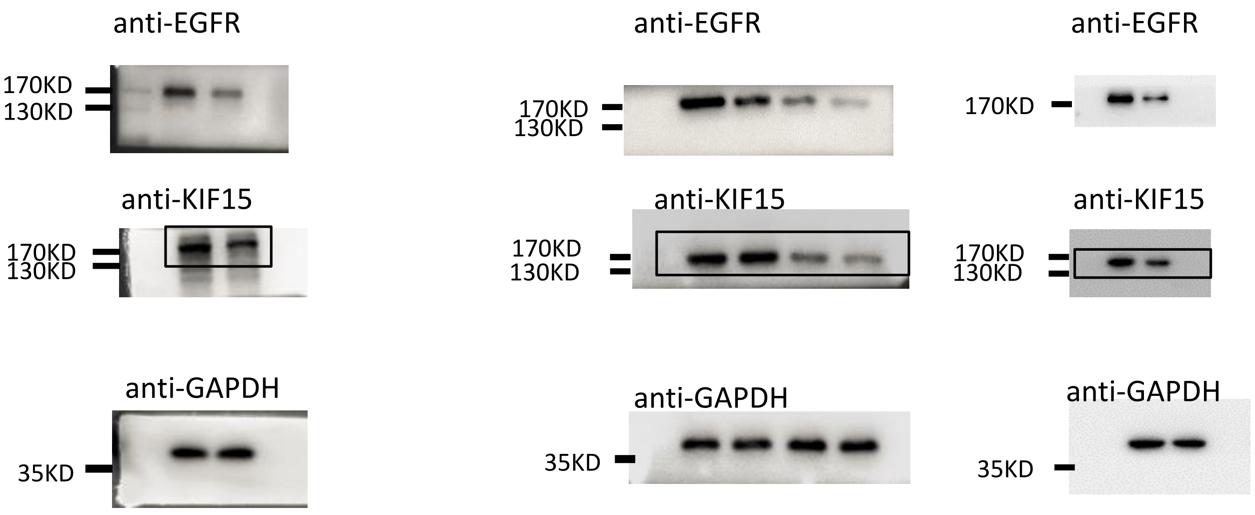

Supplementary Fig. S9. Unprocessed blot images for the western results in Supplemental Figure 1-2.
