# Supplementary figures and images for: KIF15 Promotes Progression of Castration Resistant Prostate Cancer by Activating EGFR Signaling Pathway
Source: Front Oncol. 2021 Nov 4;11:679173. doi: 10.3389/fonc.2021.679173 (PMC8599584; doi:10.3389/fonc.2021.679173)

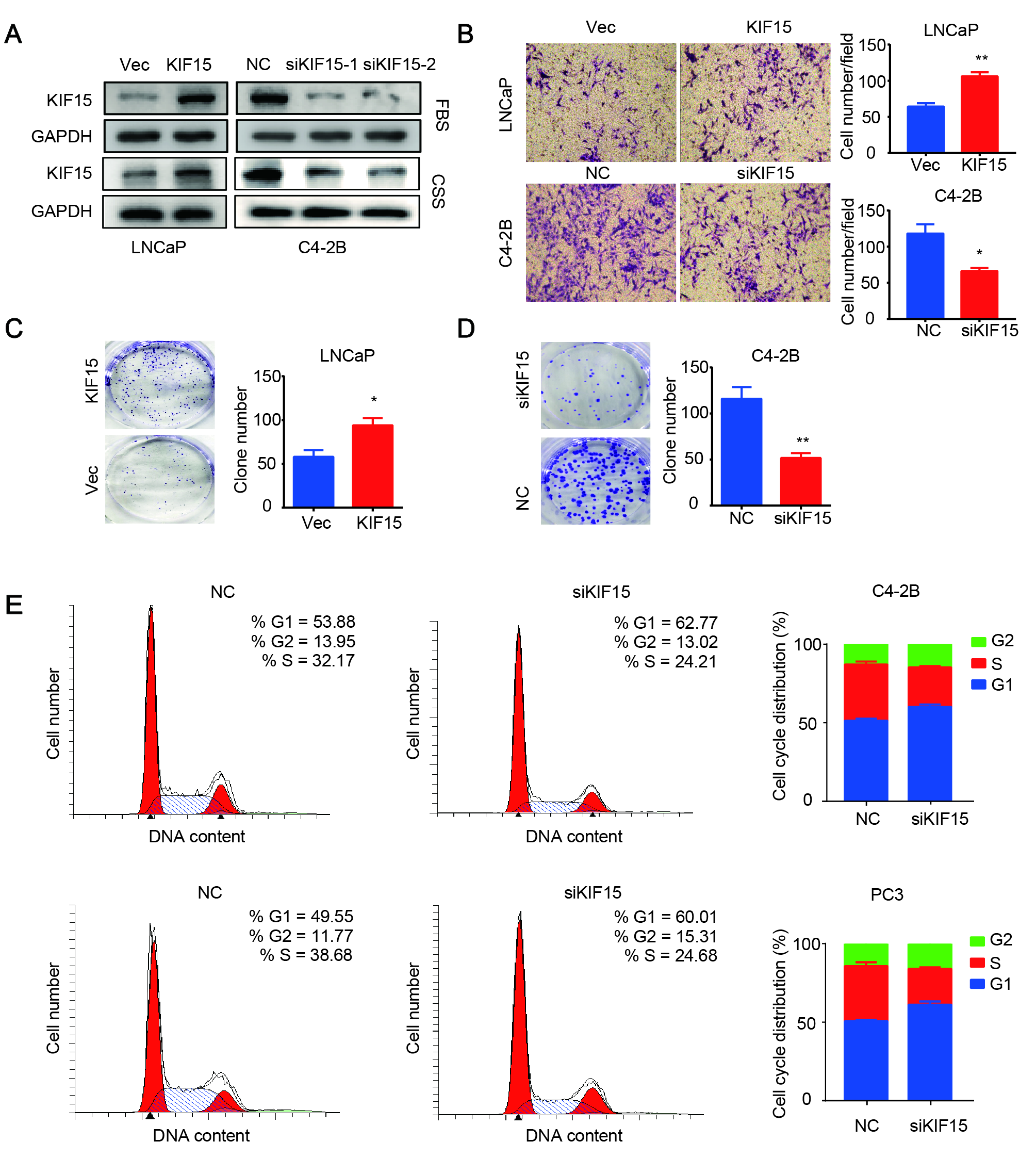

Supplement: Supplementary Figure 1 — KIF15 promotes proliferation and migration of prostate cancer cells in vitro. (A) Western blot analysis of KIF15 protein levels in indicated PCa cells. LNCaP and C4-2B cells were cultured in FBS (RPMI-1640 supplemented with 10% fetal bovine serum) medium or CSS (phenol red-free RPMI-1640 supplemented with 10% charcoal-stripped fetal bovine serum) medium for 48 hours. These cells were transfected with KIF15 expression plasmid for 48 hours (LNCaP cells) or corresponding siRNA for 72 hours (C4-2B cells), then were collected and lysed for Western blot assay. Vec, empty vector; NC, negative control. (B) Migration ability of indicated cells determined by transwell assays. Left panel: representative images of cell migration. Right panel: quantitative results of migration assays from triplicate experiments. *P <0.05, **P < 0.01. (C, D) Cell proliferation determined by colony formation assays. Data shown are means ± SEM of triplicate wells and are representative of at least three replicate experiments. Comparisons between groups were analyzed using t‐tests (two‐sided). *P <0.05, **P < 0.01. (E) Effects of KIF15 on the cell cycle determined by flow cytometry. Percentage of cells in G1, S and G2 phases are shown. C4-2B and PC3 cells were transfected with corresponding siRNA for 48 hours, then were harvested, and stained with propidium iodide dye for flow cytometric analysis of cell cycle distribution. [file Image_1.tif]

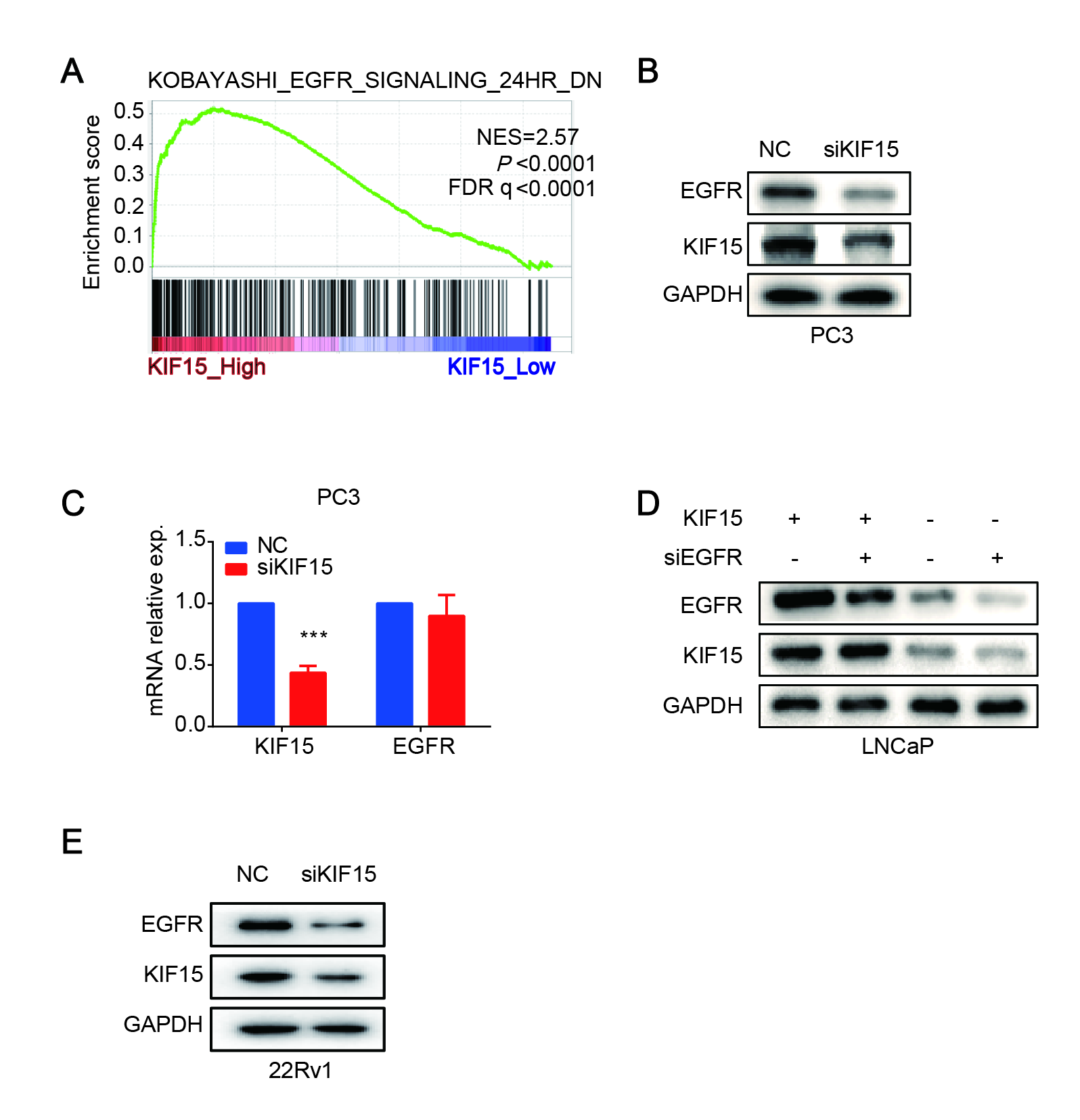

Supplement: Supplementary Figure 2 — Relationship between KIF15 and EGFR level in prostate cancer cells. (A) GSEA analysis of EGFR signatures (down-regulated after treatment with EGFR inhibitor) from a microarray dataset (GSE2443) that profiled with KIF15_High (the highest 2 samples) or KIF15_Low (the lowest 2 samples) expression. NES = 2.57; P < 0.0001, FDR q < 0.0001. (B, C) The protein (B) and mRNA (C) expression of EGFR determined by Western blot and qRT-PCR analysis after KIF15 transient knockdown in PC3 cells. PC3 cells were transfected with corresponding siRNA for 72 hours, then were harvested and lysed for Western blot assay (B). PC3 cells were transfected with corresponding siRNA for 48 hours. The total RNA was extracted, and the mRNA levels of KIF15 and EGFR were then determined by qRT-PCR (C). *P <0.05, **P < 0.01, ***P <0.001. (D) EGFR and KIF15 protein expression levels examined by Western blot in LNCaP cells. LNCaP cells were transiently transfected with the indicated expression plasmids and/or siRNA for 48 hours. The cells were then harvested and lysed for Western blot assay. (E) EGFR and KIF15 protein expression levels examined by Western blot after KIF15 siRNA knockdown in 22Rv1 cells. 22Rv1 cells were transiently transfected with corresponding siRNA for 72 hours. The cells were then harvested and lysed for Western blot assay. [file Image_2.tif]
